# Advanced Dialogues: From Laboratory to Clinics: Plant Cell‐Based Affordable Biologics

**DOI:** 10.1002/ggn2.202500045

**Published:** 2025-08-09

**Authors:** Henry Daniell

**Affiliations:** ^1^ Department of Basic and Translational Sciences School of Dental Medicine University of Pennsylvania Philadelphia PA 19104‐6030 USA

## 1 Introduction

My research group at the University of Pennsylvania School of Dental Medicine focuses on noninvasive and affordable delivery of recombinant proteins. Although biologics have been used in the clinic for more than eight decades, they are mostly unaffordable, thereby limiting their access to a large global population. The high cost is largely due to their production in cell culture systems (bacteria, yeast, CHO cells) requiring prohibitively expensive fermentation systems, purification of host cell proteins (>99%) to minimize allergic reactions, and instability of purified proteins requiring cold chain/transportation and invasive delivery through injections. Therefore, my lab pioneered the approach to develop recombinant proteins in edible plant cells that could be delivered orally via capsules or topically using chewing gums, eliminating the need for fermentation, purification, or cold chain. FDA approval of biologics bioencapsulated in plant cells has demonstrated a dramatic decrease in the cost of drugs (<5%) and a fraction of the regulatory cost for launching new drugs. Some of the recent advances are discussed in this editorial.

## 2 Overcoming the Biologics Barrier: Cost, Delivery, and Global Access

Biologics are unavailable or unaffordable for a large majority of the global population because of the way they are produced and delivered. The estimated average cost to develop a new biological product is ≈$2.6 billion.^[^ ^1^ ^]^ Among FDA‐approved biologics since 2015, >90% are injectable drugs, which are produced in prohibitively expensive fermentation systems, requiring purification and a cold chain for storage and transportation.^[^ ^2^, ^3^, ^4^ ^]^ These challenges became quite evident when only 2.2% of COVID‐19 vaccines were available for low‐income countries, and 19 million doses of mRNA vaccines were discarded in Africa due to a lack of cold chain.^[^ ^5^ ^]^ While oral or topical drugs are highly preferred by patients because of their affordability and convenience, only two oral and four topical biologic drugs were approved by the FDA since 2015,^[^ ^2^, ^3^ ^]^ probably because of regulatory guidelines developed over eight decades that are built on cell culture‐based production of biologics and injectable delivery systems.

Strikingly, the per capita prescription of drug spending in the U.S. is the highest in the world. The interquartile range of biological product prices ranged from $18861 to $288759 between 2008 and 2021.^[^ ^2^, ^3^, ^4^, ^5^, ^6^ ^]^ However, the cost of Palforzia 360 capsules with peanut cells (annual dose) is <3% (≈$2500) of the median annual price of biologics ($84508).^[^ ^2^, ^3^ ^]^ This median price excludes prohibitively expensive gene therapy drugs. Hemophilia A drug Roctavian costs $2.9 million per patient (WSJ June 29, 2023), and hemophilia B Hemgenix costs $3.5 million per patient.^[^ ^7^ ^]^ Therefore, advancing affordable biologics presents one of the greatest challenges and opportunities for health equity.

Insulin has been used in the clinic for eight decades and yet limitations of the truncated form or injectable delivery have not yet been addressed. Insulin injections delivered to the peripheral circulation are a key contributor to hypoglycemia and associated cardiac autonomic neuropathy.^[^ ^8^ ^]^ Targeting insulin to the liver via oral delivery reduces hypoglycemia.^[^ ^9^ ^]^ Frequent insulin injections contribute to treatment non‐adherence, leading to poor health outcomes.^[^ ^10^ ^]^ Cost‐related insulin rationing in developed countries has serious health consequences.^[^ ^11^ ^]^ Therefore, there is an urgent need to develop non‐invasive methods of oral or topical drug delivery.

Beyond affordability and patient compliance challenges due to invasive drug delivery, injectable proteins face several other challenges. One of the most common challenges is anti‐drug antibodies, especially in recombinant protein injections to treat hemophilia or lysosomal storage diseases.^[^ ^12^ ^]^ Indeed, gene therapy for the treatment of hemophilia excludes patients with preexisting antibodies. Oral immunotherapies have been developed, approved by the FDA, and are used in the clinic against food allergens, and this provides new opportunities to develop tolerance against injected protein drugs.^[^ ^2^, ^12^ ^]^ Advances in Generative Artificial Intelligence, accelerating drug discovery and predicting novel molecules, biomarkers, and diagnoses underscore the need for rapid drug production and delivery methods.

## 3 Cross‐Kingdom Gene Expression: Translating Plant Platforms into Biopharmaceutical Innovation

In early investigations, I developed a foreign gene expression system in chloroplasts using reporter genes.^[^ ^13^, ^14^ ^]^ However, very soon I realized that university laboratory research could go beyond basic science, and so I utilized this novel approach to engineer desired agronomic traits and improve plants by conferring resistance to herbicides, insects, or protection against abiotic stress. The ability to express high levels of these proteins (with thousands of transgene copies in each plant cell) and containment of transgene escape via pollen through maternal inheritance of engineered chloroplast genomes caught the attention of leading journals in the field^[^ ^15^, ^16^, ^17^, ^18^ ^]^ and resulted in numerous news media articles, journal covers, and editorials. Although it was exciting to see insecticidal protein crystals inside chloroplasts in electron micrographs, exceeding 50% of the total leaf protein, I realized that such high levels of expression are not necessary because commercial GM crops were successfully deployed even with <1% of the total leaf protein. Therefore, I utilized this platform technology to produce human therapeutic proteins in chloroplasts to enhance their accessibility and affordability. I am delighted that several of these products are now receiving FDA approvals for evaluation in human clinical trials.^[^ ^2^, ^4^, ^19^, ^20^, ^21^, ^22^, ^23^ ^]^ However, this multidisciplinary approach requires interdisciplinary knowledge of several fields, including plant, human, and animal biology in healthy and disease conditions, genomes, genetics, microbiome, physiology, biochemistry, and immunology approaches to address infectious diseases or metabolic disorders.

In depth knowledge and understanding of genetics and genomics are essential to push boundaries of biotechnology. For example, our early investigations on the expression of human blood proteins in chloroplasts were unsuccessful because of differences in their genomes and protein synthetic machinery. So, the first step was to sequence several chloroplast genomes and understand codon usage/hierarchy of highly expressed genes.^[^ ^23^, ^24^ ^]^ Utilizing knowledge from hundreds of sequenced chloroplast genomes, we developed an algorithm to convert human genes into highly expressed chloroplast genes and demonstrated expression and assembly of the largest human blood protein in chloroplasts.^[^ ^25^, ^26^ ^]^ Codon‐optimized human insulin gene can now be expressed in chloroplasts up to 70% of the lettuce leaf protein, with proper folding and functionality. Major advantages of plant‐cell‐based expression include complete elimination of prohibitively expensive cell culture/fermentation systems, purification, cold storage/transportation, and sterile injections. Human therapeutic proteins, are stable in freeze‐dried plant cells, are stable for many years when stored at ambient temperature and meet FDA regulatory requirements for safety, efficacy, and oral drug delivery.^[^ ^2^, ^3^, ^4^ ^]^ Again, understanding the interdisciplinary fields of plant, animal and human genetics and genomics is essential to tackle such complex biotechnology challenges.

## 4 The Quiet Joys of Recognition: What Really Matters in Science

Looking back, the most rewarding moments were receiving late‐night emails from collaborators at the National Institutes of Health or USAMRID stating that “all immunized animals that we shipped survived anthrax or plague aerosol challenge.” It is quite exciting that the vaccines we developed in plants were effective against pathogen challenge. Recognition by peers is also rewarding, especially when they were received unexpectedly. For example, when I was inducted as a Fellow of AAAS, the chair of the nomination committee was unhappy that I wasn't even a member. I had to pay for a Science journal subscription before I received this recognition at the AAAS Boston meeting, 2007. Likewise, my invitation to go to Rome in 2004 to receive the recognition of a Fellow of the oldest National Academy of Sciences in the globe was sent to the wrong addresses. I trashed the envelope, thinking that it was junk mail with several misdelivered stamps. Only later I realize that I was indeed the fourteenth American member in the 250‐year history of this academy, and Benjamin Franklin was the first American. I am now an endowed professor at the University of Pennsylvania, founded by Ben Franklin. However, these days, newly emerging AI tools like Scholar GPS rank provide the least biased quantitative measures of scholarship. Again, I was surprised to be ranked first or in the top ten globally in several fields, including genetic engineering and biopharmaceuticals. However, I encourage my mentees to enjoy recognition when they are received but that shouldn't drive their research ambition. Evaluation of the proposed hypothesis and observing the results of the experimental design are the greatest long‐lasting rewards.

## 5 Advice to Young Scientists: Think Beyond Your Field, Work Across Systems

Based on my career, one key guidance I would offer is to look for multidisciplinary collaboration opportunities to advance one's career. This requires research beyond one's area of expertise or comfort zone. Collaboration between clinicians and basic scientists enhances understanding of mechanistic aspects and the development of treatment options. Let me illustrate this one specific example. One should feel comfortable cloning genes from bacteria, yeast, fungal, human, and animal genomes to explore biotechnology applications. For example, oral cancer is initiated by HPV, anaerobic bacteria (*F. nucleatum*, *P. gingivalis*), and therefore, one needs to understand different genomes. Post surgery, after radiation therapy, when salivary cells are damaged, a decrease in saliva increases yeast colonization. So, one should understand the *Candida albicans* genome and cell wall structure to develop enzymes for disruption. So, oral cancer can't be treated without knowledge of the genomes of viruses, bacteria, and yeast in the oral cavity and host cell responses. Likewise, knowledge of the gut microbiome is essential not only for drug delivery but also to distinguish disease from a healthy microbial environment.

## 6 Shaping Research Ecosystems: Journals, Industry, and the Public Sphere

Serving as the founding editor (2002) and Editor‐in‐Chief (2012–2022) of Plant Biotechnology Journal (PBJ) has been one of the most challenging and rewarding experiences in my career. I certainly encouraged multidisciplinary articles that combined plant, animal, and human biology aspects. However, it was challenging to find multidisciplinary expertise within the same reviewer, and therefore, I sent manuscripts to different reviewers to review different sections of the same manuscript. Likewise, I encouraged special issues on genome editing before other journals recognized their importance, and now PBJ is a leading journal in this field, and genome‐edited crops are receiving regulatory approval around the globe. Therefore, I promoted a combination of basic and translational aspects of genomes and genetics. It was quite rewarding to see a young journal surpass hundred‐year‐old journals in ranking and citations. I am delighted to see several of my mentees serving the scientific community in similar roles as Executive Editors or Editors‐in‐Chief in plant science or medical journals.

Investigators must utilize knowledge gained from basic science to real‐world applications because most research funding is received from taxpayers. There is a misconception that universities are ivory towers, to focus on fundamental science and industries should focus on translational research. Although I receive most of my funding from federal agencies, I have also been funded for several decades by pharmaceutical companies like Novo Nordisk, Shire, Takeda, Johnson & Johnson, Bayer, startup companies and foundations, including Gates Foundation, Bayer Hemophilia Foundation, and American Diabetes Association, American Heart Association. Industries and foundations fund mostly translational research projects. When mentees are trained in multidisciplinary research and interact with industry, they learn valuable lessons in regulatory approval, documentation, rigor, and reproducibility of observed results, essential to launching products and benefiting the global communities.

These days, journalists play a key role in writing news stories that bring breakthrough innovations to the attention of investors. I realized the power of social and news media outlets of well‐written news articles, without exaggeration and scientific jargon, reaching millions of Twitter exchanges or news stories in more than a hundred global languages. Several journals track Altmetric scores and provide authors with global outreach to evaluate the timely impact of scientific publications. Cross‐sector partnerships require coordination of funding agencies, industries, foundations, and publishers. It takes an entire village to change the status quo and break the traditional disciplinary boundaries and silos.

## 7 Power of International Collaborations

Science has no national boundaries. In the past four decades, I have had the privilege of hosting investigators in my lab from all continents. I introduced Associate Editors from all continents when I served as the Editor in Chief of PBJ, which dramatically enhanced journal citation and ranking, especially due to social media exchanges of published articles in different global languages. I also worked hard to convert PBJ to an Open Access Journal so that readers around the globe could have free access, without paying a journal subscription. Although many of my mentees pursue successful careers in academia or industry in the United States, those who return to their home countries have established wonderful research programs. This summer, I was quite impressed by the accomplishments of my mentee, Dr. Shuangxia Jin, Dean at Huazhong Agricultural University, Wuhan, when I gave a keynote address in the 120th Anniversary of Guizhou Academy of Agricultural Sciences, organized by Dr. Jin. The following week, I visited India to give a keynote speech organized by another mentee, Dr. Shashi Kumar, at the United National Institute (ICGEB, New Delhi), and thrilled to observe algae from his lab were tested by the first Indian astronaut in space station. These are great examples of international collaborations that require multidisciplinary skills.

## 8 Beyond Balance: Work and Life as One

This topic is discussed a lot more these days, with the misconception that one negatively impacts the other. Indeed, forty years ago, when I moved to the US, many of my postdoctoral colleagues worked late nights, during weekends, and scheduling lab meetings during weekends, especially during the winter months, was challenging. Most of my colleagues never complained and have successful professional and personal lives. Several of my mentees who had children during their graduate or postdoctoral studies had excellent time management skills and are highly successful in their personal and professional lives. In contrast, those who limited their time in the lab due to work‐life balance changed jobs frequently, with an uncertain future. From my personal experience, juggling the responsibility of Editor in Chief of a major journal, reviewing thousands of manuscripts, writing grants to support a very large number of investigators in my lab, responsibilities as founder of biotech companies, prosecution of >100 patents, FDA regulatory approval documentation, teaching and professional travel couldn't be done within 40 h in any week. Even when I am on vacation, I keep thinking of projects, ideas, and outcomes of lab investigations. Therefore, my life and work are deeply integrated and inseparable.

## Conflict of Interest

The authors declare no conflict of interest. However, the author is an inventor or coinventor on a large number of patents and has been supported by several pharmaceutical companies in the past. List of patents are publicly available in Scholar GPS or Google Scholar links provided below. https://scholargps.com/scholars/82094026790000/henry-daniellhttp://scholar.google.com/citations?user=7sow4jwAAAAJ&hl=en

## References

[1] J. A. DiMasi , H. G. Grabowski , R. W. Hansen , J. Health Econ. 2016, 47, 20. 26928437 10.1016/j.jhealeco.2016.01.012

[2] H. Daniell , R. J. Kulcharo , R. W. Herzog , M. Kulis , K. W. Leong , Nat. Biotechnol. 2023, 41, 1186. 37550435 10.1038/s41587-023-01899-1

[3] R. J. Kulchar , R. Singh , S. Ding , E. Alexander , K. W. Leong , H. Daniell , Biomaterials 2023, 302, 122312. 37690380 10.1016/j.biomaterials.2023.122312 PMC10840840

[4] H. Daniell , G. Wakade , S. K. Nair , R. Singh , S. A. Emanuel , B. Brock , K. B. Margulies , Pharmaceutics 2024; 16, 3325219. 10.3390/pharmaceutics17010012 PMC11768411 39861664

[5] M. M. Loemb´e , J. N. Nkengasong , Immunity 2021, 54 1353. 34260880 10.1016/j.immuni.2021.06.017 PMC8276532

[6] B. N. Rome , A. C. Egilman , A. S. Kesselheim , 2022 JAMA, J. Am. Med. Assoc. 327, 2145. 10.1001/jama.2022.5542 PMC9175077 35670795

[7] M. Naddaf , Nature 2022, 612, 388. 36474054 10.1038/d41586-022-04327-7

[8] A. J. Flatt , A. J. Peleckis , C. Dalton‐Bakes , H.‐L. Nguyen , S. Ilany , A. Matus , S. K. Malone , N. Goel , S. Jang , J. Weimer , I. Lee , M. R. Rickels , Diabetes Technol. Ther. 2023, 25, 10.1089/dia.2022.0506. PMC10171955 36763336

[9] D. S. Edgerton , M. C. Moore , J. M. Gregory , G. Kraft , A. D. Cherrington , Am. J. Physiol. Endocrinol. Metab. 2021, 320 E891. 33813879 10.1152/ajpendo.00628.2020 PMC8238128

[10] P. McEwan , J. Baker‐Knight , B. Ásbjörnsdóttir , Y. Yi , A. Fox , R. Wyn , Eur. J. Health Econ. 2023, 24, 187. 35526173 10.1007/s10198-022-01470-w PMC9080344

[11] M. Fang , E. Selvin , JAMA, J. Am. Med. Assoc. 2023, 329, 1700. 10.1001/jama.2023.5747 PMC10061307 36988971

[12] J. S. S. Butterfield , X. Li , S. Arisa , K. C. Kwon , H. Daniell , R. W. Herzog , Cell. Immunol. 2023, 391, 104742. 37423874 10.1016/j.cellimm.2023.104742 PMC10529677

[13] H. Daniell , B. A. McFadden , Proc. Natl. Acad. Sci. USA 1987, 84, 6349. 3114748 10.1073/pnas.84.18.6349 PMC299073

[14] H. Daniell , J. Vivekananda , B. Nielsen , G. Ye , K. K. Tewari , J. C. Sanford , Proc. Natl. Acad. Sci. USA 1990, 87, 88. 2404285 10.1073/pnas.87.1.88 PMC53205

[15] M. Kota , H. Daniell , S. Varma , S. V. Garczynski , F. Gould , W. J. Moar , Proc. Natl. Acad. Sci. USA 1999, 96, 1940. 10.1073/pnas.96.5.1840 PMC26698 10051556

[16] H. Daniell , R. Datta , S. Varma , S. Gray , S.‐B. Lee , Nat. Biotechnol. 1998, 16, 345. 9555724 10.1038/nbt0498-345 PMC5522713

[17] B. DeCosa , W. Moar , S. B. Lee , M. Miller , H. Daniell , Nat. Biotechnol. 2001, 19, 71. 11135556 10.1038/83559 PMC4560096

[18] H. Daniell , Nat. Biotechnol. 2002, 20, 581. 12042861 10.1038/nbt0602-581 PMC3471138

[19] H. Daniell , R. Singh , V. Mangu , S. K. Nair , G. Wakade , N. Balashova , Biomaterials 2023, 298, 122142. 37148757 10.1016/j.biomaterials.2023.122142 PMC10219636

[20] H. Daniell , Y. Guo , R. Singh , U. Karki , R. J. Kulchar , G. Wakade , J.‐M. Pihlava , H. Khazaei , G. H. Cohen , Mol. Ther. 2025, 33, 184. 39663701 10.1016/j.ymthe.2024.12.008 PMC11764783

[21] H. Daniell , S. K. Nair , N. Esmaeili , G. Wakade , N. Shahid , P. K. Ganesan , M. R. Islam , A. Shepley‐Mctaggart , S. Feng , E. N. Gary , Mol. Ther. 2022, 30, 1966. 34774754 10.1016/j.ymthe.2021.11.008 PMC8580552

[22] H. Daniell , S. K. Nair , H. Guan , Y. Guo , R. J. Kulchar , M. D. Torres , M. Shahed‐Al‐Mahmud , G. Wakade , Y.‐M. Liu , A. D. Marques , Biomaterials 2022, 288, 121671. 35953331 10.1016/j.biomaterials.2022.121671 PMC9290430

[23] H. Daniell , H.‐T. Chan , E. K. Pasoreck , Annu. Rev. Genet. 2016, 50, 595. 27893966 10.1146/annurev-genet-120215-035349 PMC5496655

[24] H. Daniell , C.‐S. Lin , M. Yu , W.‐J. Chang , Genome Biol. 2016, 17, 134. 27339192 10.1186/s13059-016-1004-2 PMC4918201

[25] K.‐C. Kwon , H.‐T. Chan , I. R. León , R. Williams‐Carrier , A. Barkan , H. Daniell , Plant Physiol. 2016, 172, 62. 27465114 10.1104/pp.16.00981 PMC5074611

[26] K.‐C. Kwon , A. Sherman , W.‐J. Chang , A. Kamesh , M. Biswas , R. W. Herzog , H. Daniell , Plant Biotechnol. J. 2018, 16, 1148. 29106782 10.1111/pbi.12859 PMC5936678

